# Vocal biomarkers for geriatric health assessment: a scoping review protocol

**DOI:** 10.1136/bmjopen-2025-109480

**Published:** 2026-02-23

**Authors:** Mehrdad Amir-Behghadami, Kamal Gholipour, Zeinab Mohammadzadeh

**Affiliations:** 1 Student Research Committee (SRC), Tabriz University of Medical Sciences, Tabriz, Iran; 2 Iranian Center of Excellence in Health Management, Department of Health Service Management, School of Management and Medical Informatics, Tabriz University of Medical Sciences, Tabriz, Iran; 3 Department of Health Service Policy& Management, Tabriz Health Services Management Research Center, School of Management and Medical Informatics, Tabriz University of Medical Sciences, Tabriz, Iran

**Keywords:** Health informatics, Information management, Information technology, Telemedicine, GERIATRIC MEDICINE, Artificial Intelligence

## Abstract

**Abstract:**

**Introduction:**

Global ageing populations require accessible, non-invasive tools for early detection and monitoring of neurological chronic and neurodegenerative diseases. Current diagnostic methods face limitations including invasiveness, high costs and infrequent clinical assessments. The human voice has emerged as a promising digital biomarker, with vocal characteristics reflecting physiological and cognitive changes associated with conditions like dementia and Parkinson’s disease. While artificial intelligence (AI) and machine learning have enabled sophisticated vocal analysis, the literature remains fragmented without comprehensive synthesis. This scoping review protocol delineates a systematic approach to collate and synthesise existing research on the application of AI-driven audio biomarkers for the detection and management of neurological diseases (eg, cognitive decline, Parkinson’s disease, Alzheimer’s, dementia and depression) in older adults aged 65 years and above.

**Methods and analysis:**

This scoping review will be conducted in accordance with the PRISMA-ScR (Preferred Reporting Items for Systematic Reviews and Meta-Analyses extension for Scoping Reviews) guidelines and the methodological framework proposed by Arksey and O’Malley, incorporating recent methodological advancements. The eligibility criteria for study selection will be formulated using the PCC (Population, Concept, Context) framework. A comprehensive literature search will be performed across several electronic databases, including PubMed/MEDLINE, Scopus, Web of Science, IEEE Xplore, Embase, Compendex, CINAHL, Scientific Information Database (SID), Magiran, IranMedex and Barakat Knowledge Network System (BKNS). The search will encompass peer-reviewed articles published in Persian and English from 1 January 2012 to 31 March 2026. Two independent reviewers will screen titles, abstracts and full texts and extract data according to the predefined PCC-based eligibility criteria. Discrepancies will be resolved through discussion or, if necessary, by consultation with a third reviewer. The results will be synthesised and presented narratively, accompanied by summary tables, charts and figures to address each research question.

**Ethics and dissemination:**

The Research Ethics Committee of Tabriz University of Medical Sciences approved the protocol for this scoping review (approval number: IR.TBZMED.VCR.REC.1404.223). They concluded that since the review involves only analysis of existing literature without direct patient involvement or clinical procedures, it meets the relevant ethical standards. Results from the review will be shared through peer-reviewed journals and conference presentations to provide valuable insights for researchers, clinicians and policymakers on the use of audio-based biomarkers in older adults.

**PROSPERO registration number:**

Not registered.

**STRENGTHS AND LIMITATIONS OF THIS STUDY**

- A comprehensive search protocol will be developed with a medical librarian to ensure extensive database coverage.
- Dual independent screening and data extraction will be implemented to reduce selection bias.
- The study protocol will be designed with input from a multidisciplinary team of clinicians, artificial intelligence experts and scoping review specialists.
- As a scoping review, formal quality assessment and risk of bias evaluation of included studies will not be conducted.

## Introduction

 The global population is undergoing an unprecedented demographic shift characterised by rapid ageing, with projections indicating that approximately 22% of individuals will be aged 60 years or older by 2050.^1^ This transition strains healthcare systems, especially for early detection of age-related neurological conditions such as cognitive decline, Parkinson’s disease and depression—excluding non-neurological voice-affecting issues like respiratory disorders to sharpen focus on neurological biomarkers. Traditional diagnostic methods often rely on invasive procedures or subjective assessments, which may delay timely intervention.^2 3^ Advances in artificial intelligence (AI) and machine learning (ML) enable non-invasive voice analysis, leveraging cost-effective, scalable acoustic features to detect subtle neurological changes in older adults.^4^

Voice analysis excels for geriatric neurological assessment due to its remote feasibility—ideal for mobility-limited individuals—its sensitivity to early cognitive and motor alterations, and its potential for preclinical detection.^4^,^6^

Yet, the literature reveals a critical gap: marked heterogeneity in voice recording protocols, feature extraction and AI/ML algorithms across studies of neurological conditions, coupled with scant real-world validation outside laboratories, impedes comparability, standardisation and clinical translation.^7 8^ Ethical issues—data privacy, bias, digital divides—and implementation barriers like governance remain underexplored, particularly their ties to core questions on methodological rigour, performance in diverse settings and equitable deployment for geriatric care.^9 10^

Studies affirm voice biomarkers’ promise for neurological disorders: acoustic/linguistic features detect mild cognitive impairment (MCI) and Alzheimer’s with high accuracy;^11^ ML models track Parkinson’s progression; acoustic markers like reduced pitch variability aid depression screening; and vocal frailty correlates predict physical decline in ageing—though all require synthesis amid inconsistencies.^12 13^

This scoping review protocol maps evidence on AI-enabled voice biomarkers exclusively for these neurological domains in older adults. It synthesises methodologies, performance, gaps and patterns to guide standardisation and clinical pathways. Simultaneously, it interrogates ethical/implementation linkages to review aims, informing privacy safeguards, bias mitigation and governance for inclusive adoption—ultimately advancing geriatric health outcomes.

## Methods and analysis

### Review method

The summary of this protocol will be reported in accordance with the Preferred Reporting Items for Systematic Reviews and Meta-Analyses (PRISMA) Protocols statement^14^ (see online supplemental additional 1). The forthcoming scoping review will adhere to the reporting guidelines outlined in the PRISMA extension for Scoping Reviews (PRISMA-ScR).^15^ Registration of this protocol in PROSPERO will not be feasible, as PROSPERO does not currently accept scoping reviews.

This study will be conducted following the methodological framework originally proposed by Arksey and O’Malley,^16^ alongside the guidance provided in the Joanna Briggs Institute (JBI) Reviewers’ Manual, which has been further refined by subsequent authors.^17^,^19^ Scoping reviews are designed to systematically explore the extent, range and nature of research activity on a given topic, as well as to identify existing gaps within the literature. Accordingly, this review aims to identify and map the existing evidence on the use of AI with voice biomarkers for the management of elderly patients who can be diagnosed or monitored through vocal indicators. The review process will follow six sequential stages: (1) formulation of the research question, (2) identification of relevant studies, (3) study selection, (4) data charting, (5) data analysis and reporting and (6) consultation exercise.^16^

### Stage 1: identifying the research question

#### Research question

How are audio-based biomarkers being used in the detection and management of diseases among older adults? Specifically, what are the applications of AI and ML in the detection and management of age-related diseases among older adults using voice samples as digital biomarkers?

#### Specific sub-questions

The specific research questions in this scoping review are systematically organised within six inter-related thematic topics: (1) clinical applications in diagnosis and management, (2) methodological approaches to voice data collection, (3) acoustic feature extraction and analysis, (4) AI/ML model architectures and performance metrics, (5) demographic considerations and generalisability and (6) implementation challenges and translational potential.

1. Clinical Applications
  - What are the primary clinical applications of voice biomarker analysis in older adults, including:
    - Diagnosis (eg, early detection of cognitive decline, Parkinson’s disease)?
    - Monitoring (eg, tracking disease progression or treatment response)?
    - Intervention (eg, voice-driven therapeutic tools or feedback systems)?
2. Methodologies for voice recording
  - What are the standard protocols for voice data collection in studies of older adults?
    - Tasks: Free speech, reading tasks, sustained vowels or structured interviews?
    - File formats: Lossless (WAV, FLAC) versus compressed (MP3, AAC)?
    - Equipment: Clinical-grade microphones versus consumer devices (smartphones, wearables)?
    - Environment: Controlled clinical settings versus remote/home recordings?
3. Audio features and AI/ML inputs
  - Which acoustic features (eg, pitch, jitter, speech rate, spectral tone) are most frequently used as inputs for AI/ML models in older adult populations?
  - Are there disease-specific feature patterns (eg, dysarthria in Parkinson’s vs semantic pauses in Alzheimer’s)?
4. AI/ML models and performance
  - Which AI/ML models (eg, Support Vector Machine (SVM), Convolutional Neural Network (CNN), Long Short-Term Memory (LSTM), Random Forest) demonstrate the highest efficacy for detecting/managing age-related diseases via voice?
  - What evaluation metrics (eg, accuracy, Area Under the Cruve-Receiver Operating (AUC-ROC), F1-score) are reported, and how do they vary by disease or task?
5. Demographic and clinical generalisability
  - To what extent do datasets and models account for variability in:
    - Demographics: Age, sex, ethnicity, multilingualism?
    - Comorbidities: Hearing loss, respiratory conditions or polypharmacy?
  - How does this variability impact model performance and clinical applicability?
6. Implementation barriers and facilitators
  - What are the key barriers to adoption (eg, dataset bias, regulatory hurdles, clinician scepticism)?
  - What facilitators exist (eg, telehealth integration, low-cost devices, patient acceptability)?

This scoping review will be conducted using the Population, Concept and Context (PCC) framework, as recommended by the JBI for scoping reviews, to guide the search strategy.^20^

- Population: Older adults aged 65 years and above who have or are at risk of neurological age-related neurological or neurodegenerative conditions.
- Concept: The focus is on voice and speech analysis using AI and ML techniques to extract clinically relevant biomarkers.
- Context: The scope covers the detection, diagnosis and management of neurological age-related diseases, focusing on cognitive decline, Parkinson’s disease, Alzheimer’s, dementia and depression, specifically excluding non-neurological conditions that affect the voice.

The PCC framework is more appropriate than the Patient/Problem, Intrvention, Comparison, outcome (PICO) tool for capturing a broad range of study designs related to the sub-questions, as all studies with quantitative, qualitative and mixed methodologies will be relevant for the purpose of this review.

### Inclusion criteria

#### Publication characteristics

- Peer-reviewed primary research articles published in reputable scientific journals.
- Published in English or Persian.
- Publication date: January 2012 to present.
- Study types: Qualitative research, randomised controlled trials, non-randomised studies, quantitative descriptive studies and mixed methods studies.

#### Methodological requirements

- Must employ voice recordings as primary input data.
- Must use at least one AI/ML technique (eg, SVM, neural networks, random forest).
- Must report quantitative performance metrics (eg, accuracy, AUC-ROC)

#### Population and focus

- Studies investigating adults aged ≥65 years.
- Focus on age-related neurological/neurodegenerative conditions (eg, Alzheimer’s, Parkinson’s, MCI).
- Applications must include at least one of: disease detection/diagnosis, symptom/progression monitoring or therapeutic intervention evaluation.

### Exclusion criteria

#### Publication characteristics

- Preprints, conference abstracts without full papers, non-peer-reviewed publications.
- Represent non-primary research (commentaries, letters, editorials, opinions, case reports or secondary analyses)

#### Language and date

- Articles in languages other than English/Persian.
- Published before 2012.

#### Data type

- Studies using non-vocal biomarkers (eg, electroencephalography (EEG), imaging, text transcripts).
- Research analysing non-speech sounds (eg, coughs, breathing sounds) unless complementary to voice analysis.

#### Technical approach

- Studies without AI/ML components (eg, purely statistical analyses).
- Algorithm development papers without clinical validation.

#### Disease scope

- Conditions primarily affecting voice through non-neurological mechanisms:
  - Respiratory disorders (eg, Chronic Obstructive Pulmonary Disease (COPD), laryngitis).
  - Gastrointestinal pathologies (eg, Gastroesophageal Reflux Disease (GERD)).
  - Acute infectious diseases (eg, COVID-19 voice changes).

#### Population

- Studies focused on paediatric or middle-aged adults (<65 years) without older adult subgroup analysis

### Stage 2: identification of relevant studies

The commonly used terms suggested by the authors for identifying relevant articles include AI, voice, ageing and ageing health. Following an initial search, new relevant keywords will be identified. These additional terms will be incorporated into a comprehensive search string, which will be finalised and approved by all members of the research team. A draft version of the search string is provided (see online supplemental additional file 2).

This search string will be applied to major databases including PubMed/MEDLINE, Scopus, Web of Science, IEEE Xplore, Embase, Compendex, CINAHL, Scientific Information Database (SID), Magiran, IranMedex and Barakat Knowledge Network System (BKNS) to retrieve all relevant peer-reviewed primary journal articles published between 1 January 2012 to 31 March 2026. Additionally, Google Scholar and ClinicalTrials.gov will be searched to identify grey literature.

The search strategy will primarily use Medical Subject Headings (MeSH) terms, supplemented by keywords tailored to the advanced search requirements of each database. Boolean operators AND/OR will be employed to combine and refine search terms. Filters will be applied to limit results to publications dated from 1 January 2012 to 31 March 2026. This timeframe was selected after consultation with experts in medical informatics and ageing health, reflecting the period of significant technological advancements in healthcare and accommodating the latest evidence up to the review’s completion.

### Stage 3: study selection

This study will use a systematic and rigorous process for selecting and screening studies. Following the PRISMA-ScR guidelines, the selection stages will be structured as follows: First, all retrieved studies from various scientific databases will be imported into EndNote V.8 software, where duplicates will be automatically removed. Bilingual screening in our study selection is feasible and reliable due to the authors’ multilingual proficiency and the systematic calibration of the screening process to ensure consistency and accuracy across Persian, Turkish and English-language sources. Next, two experienced researchers will independently and in parallel review all titles and abstracts carefully. Studies meeting the predefined inclusion criteria will proceed to full-text assessment. At the full-text stage, both researchers will separately analyse the complete articles and decide on their eligibility based on strict inclusion and exclusion criteria. To ensure comprehensiveness, a supplementary step will manually review the reference lists of all selected articles and studies citing them, helping to identify any relevant studies that might have been missed. In cases of disagreement between the two researchers regarding a study’s suitability, discussions will be held to resolve conflicts. If consensus cannot be reached, a third researcher will act as the final arbiter. For studies with only abstracts available, the corresponding authors will be contacted to obtain the full text if possible. All steps of this process, including reasons for study exclusions, will be meticulously documented and presented in a PRISMA flow diagram. This precise and scientific approach ensures that the review’s findings are based on the best and most relevant evidence while minimising selection bias. Full transparency will be maintained throughout, allowing other researchers to review and assess the study selection process.

### Stage 4: data charting

This study employs a rigorous, multi-step process for data extraction. Initially, a standardised data extraction form will be developed in MS Word, based on the JBI guidelines. This form will include study characteristics, research objectives, methodology, participant features, details of voice data, AI model parameters and performance evaluation criteria (eg, AUC, sensitivity, specificity reported descriptively by model type). To ensure data accuracy, two researchers will independently review 20% of the studies. Inter-rater reliability will be assessed using Cohen’s κ and percentage agreement to quantify consistency between reviewers. After confirming consistency between their results, the remaining data extraction will be conducted by a single researcher. Finally, a team of three experts will critically evaluate all extracted data to fully ensure the accuracy and validity of the findings. This systematic and layered approach maintains the quality and comprehensiveness of the data extraction process throughout the study.

### Stage 5: data analysis and reporting the results

This study aims to map patterns in voice changes while examining existing heterogeneities by systematically categorising relevant studies according to key sources of variation (eg, voice recording protocols, feature extraction methods, AI/ML algorithms, study populations and performance metrics). Eligible studies meeting the inclusion and exclusion criteria will be organised into specialised subgroups structured by primary neurological domain: (1) cognitive decline/dementia (Alzheimer’s, MCI), (2) Parkinson’s disease and (3) depression; with a dedicated section for multimorbidity/overlaps in older adults (eg, co-occurring Parkinson’s+depression studies tabulated separately) focused on the applications of AI and ML in ageing health through audio-based biomarkers. Heterogeneity will be handled descriptively by: (1) tabulating variations across subgroups, (2) narratively synthesising common patterns and divergences without meta-analysis, (3) summarising performance metrics descriptively (eg, range of reported AUC values by algorithm type) without implying comparative effectiveness across heterogeneous models and (4) highlighting implications for standardisation and future research. The findings will be presented comprehensively using various formats, including narrative analyses, comparative tables (eg, protocol variations by disease), analytical charts (eg, bar graphs of feature types used) and conceptual models (eg, flowchart mapping biomarker-to-outcome pathways across domains). This multidimensional approach enables a detailed examination of voice change patterns, identification of influencing factors and a deeper understanding of heterogeneities across studies. Each section of the results will be designed and reported in alignment with the research questions to provide a complete picture of the clinical applications of these technologies.

### Stage 6: consultation exercise

The consultation exercise in this phase will primarily be conducted internally by members of the research team as part of the analytical process to refine the scoping review findings. Initial findings may then be presented to a panel of experts in a separate study for further review and validation, specifically in the design of an AI decision support system based on voice biomarkers in ageing. This panel will include AI experts, clinicians such as physicians, geriatricians and other relevant experts to ensure a comprehensive and multidisciplinary assessment. Feedback from these stakeholders will be systematically collected and used to refine those subsequent applications, enhancing the applicability and impact of the findings.

### Patient and public involvement

None.

### Ethics and dissemination

#### Ethics

The protocol for this scoping review was approved by the Research Ethics Committee of Tabriz University of Medical Sciences (approval number: IR.TBZMED.VCR.REC.1404.223). The committee determined that the review’s objectives do not entail direct involvement of patients or clinical interventions, thereby upholding ethical requirements relevant to research based solely on literature review.

#### Dissemination

We intend to publish the findings of this scoping review in a peer-reviewed journal specialising in AI applications or voice biomarker research. Additionally, the results will be disseminated at both national and international conferences to reach a broad audience of clinical and technological researchers. The outcomes of this study will also be shared through seminars or journal club presentations at the Development and Technology Center and the Tabriz Psychiatric Research Center, both affiliated with Tabriz University of Medical Sciences, Tabriz, Iran.

## Discussion

This scoping review anticipates mapping the emerging role of audio-based biomarkers as non-invasive tools for neurological disorders in older adults, potentially identifying voice alterations linked to conditions such as Parkinson’s disease and Alzheimer’s disease.

Prior literature largely focuses on disease-specific reviews, such as those addressing respiratory conditions or frailty alone, lacking a comprehensive overview of neurological ageing-related disorders detectable via vocal biomarkers.^21^,^23^ Incorporating findings from systematic and scoping reviews reporting variable but promising diagnostic accuracy, the present protocol addresses the need for synthesis across a neurological spectrum. This broader perspective is essential to capture the complex interplay of age-related neurological diseases reflected in voice and speech changes.^22 23^

Practical implementation of vocal biomarkers for geriatric health assessment faces notable challenges, particularly in resource-limited settings where inadequate infrastructure, limited access to digital tools and deficits in interdisciplinary training may significantly hinder adoption. Such environments often lack the necessary technological foundation, including reliable internet connectivity and compatible devices, which are vital for deploying voice-based digital health solutions. Furthermore, the interdisciplinary expertise required to develop, implement and maintain these advanced AI-driven tools remains scarce, which affects integration and sustainability. Addressing these barriers necessitates targeted strategies such as capacity building through specialised training programmes, engaging diverse stakeholders including clinicians and patients in co-design processes and embedding vocal biomarker technologies within existing clinical workflows to enhance acceptability and feasibility. These approaches are critical for overcoming current limitations and ensuring effective translation of vocal biomarker innovations into routine geriatric care, particularly in underserved regions.^24^

This protocol anticipates specifying strategies for update, such as annual literature surveillance incorporating emerging AI terms (eg, generative/multimodal models), aligned with methodological guidance for dynamic fields. ^25^

This scoping review protocol will develop a comprehensive framework that informs clinical practice, policy-making and AI-driven innovation in digital health for older adults. By systematically identifying research gaps and addressing the heterogeneity in methodological approaches, this work seeks to promote the advancement of standardised protocols and tailored intervention strategies. Anticipated outcomes include enhanced understanding of early detection potential and continuous monitoring applications, supporting—but not prescribing—quality of life improvements among ageing populations.

While this scoping review protocol acknowledges the absence of a formal quality assessment of included studies and English-language restriction, which are consistent with scoping review methodology, further limitations have been identified to strengthen its rigour. The potential for publication bias exists due to the non-publication of negative or inconclusive findings in the rapidly growing field of digital biomarkers, which could skew available evidence. Additionally, the evolving regulatory landscape surrounding digital health technologies and voice biomarkers, including variable approval pathways and data privacy regulations, may impact clinical applicability and policy translation. These dynamic factors warrant ongoing surveillance and consideration when interpreting review results to ensure responsible implementation.

## Supplementary material

10.1136/bmjopen-2025-109480 online supplemental file 1

10.1136/bmjopen-2025-109480 online supplemental file 2
