# Supplementary material for: Vocal biomarkers for geriatric health assessment: a scoping review protocol
Source: BMJ Open. 2026 Feb 23;16(2):e109480. doi: 10.1136/bmjopen-2025-109480 (PMC12931537; doi:10.1136/bmjopen-2025-109480)
Supplement: online supplemental file 2 [file bmjopen-16-2-s002.docx]

| Searches run: …………2025 | |
| --- | --- |
| Publication date filter: 1 January 2012–2025 | |
| Language filter: None | |
| Search | Results |
| PubMed | 00 |
| **#1: Keywords related to artificial intelligence or machine learning** |  |
| ("Artificial Intelligence"[Mesh] OR "artificial intelligence"[tiab] OR "machine learning"[tiab] OR "deep learning"[tiab] OR "computational intelligence"[tiab] OR "computer reasoning"[tiab] OR "computer vision system"[tiab] OR "computer vision systems"[tiab] OR "transfer learning"[tiab] OR "hierarchical learning"[tiab] OR "learning from labeled data"[tiab] OR "support vector network"[tiab] OR "support vector networks"[tiab] OR "support vector machine"[tiab] OR "support vector machines"[tiab] OR "ambient intelligence"[tiab] OR "automated reasoning"[tiab] OR "computer heuristics"[tiab] OR "cognitive technology"[tiab] OR "cognitive technologies"[tiab] OR "cognitive computing"[tiab] OR "cognitive robotics"[tiab] OR "optical character recognition"[tiab] OR "robotic process automation"[tiab] OR "machine intelligence"[tiab] OR "artificial superintelligence"[tiab] OR "artificial general intelligence"[tiab] OR "machine reasoning"[tiab] OR "automated inference"[tiab] OR "heuristic algorithm"[tiab] OR "heuristic algorithms"[tiab] OR metaheuristic*[tiab] OR meta-heuristic*[tiab] OR "data mining"[tiab] OR "neural network"[tiab] OR "neural networks"[tiab] OR "neural networking"[tiab] OR "feature learning"[tiab] OR "feature extraction"[tiab] OR "Bayesian learning"[tiab] OR "Bayesian inference"[tiab] OR "multicriteria decision analysis"[tiab] OR "unsupervised learning"[tiab] OR "semi-supervised learning"[tiab] OR "semi supervised learning"[tiab] OR "ANN analysis"[tiab] OR "ANN analyses"[tiab] OR "ANN method"[tiab] OR "ANN methods"[tiab] OR "ANN model"[tiab] OR "ANN models"[tiab] OR "ANN modeling"[tiab] OR "ANN methodology"[tiab] OR "ANN methodologies"[tiab] OR "artificial NN"[tiab] OR "ANN technique"[tiab] OR "ANN techniques"[tiab] OR "ANN output"[tiab] OR "ANN outputs"[tiab] OR "ANN approach"[tiab] OR "network learning"[tiab] OR "random forest"[tiab] OR "relevance vector machine"[tiab] OR "relevance vector machines"[tiab] OR "online analytical processing"[tiab] OR "sentiment analysis"[tiab] OR "sentiment analyses"[tiab] OR "opinion mining"[tiab] OR "sentiment classification"[tiab] OR "sentiment classifications"[tiab] OR "fuzzy logic"[tiab] OR "natural language processing"[tiab] OR "expert system"[tiab] OR "expert systems"[tiab] OR "biological ontology"[tiab] OR "biological ontologies"[tiab] OR "biomedical ontology"[tiab] OR "biomedical ontologies"[tiab] OR "computer simulation"[tiab] OR "computer simulations"[tiab] OR "Multidimensional Voice Program"[tiab] OR MDVP[tiab] OR "k-nearest neighbor"[tiab] OR "supervised learning algorithm"[tiab] OR "swarm intelligent"[tiab] OR "Swarm intelligence"[tiab] OR "firefly algorithm"[tiab] OR bootstrap*[tiab]) | 00 |
| **#2: Keywords related to voice biomarkers** |  |
| (voice[Mesh] OR voice*[tiab] OR speech*[tiab] OR acoustic*[tiab] OR phonat*[tiab] OR vox[tiab] OR language*[tiab] OR linguistic*[tiab] OR speak*[tiab] OR sing*[tiab] OR vocal*[tiab] OR respirat*[tiab] OR articulat*[tiab] OR prosody[tiab] OR pitch[tiab] OR "fundamental frequency*"[tiab] OR f0[tiab] OR "disturbance index"[tiab] OR jitter*[tiab] OR shimmer*[tiab] OR "vocal intensity"[tiab] OR "acoustic voice quality index"[tiab] OR AVQI[tiab] OR "speech to noise ratio*"[tiab] OR "sound pressure level*"[tiab] OR "cepstral peak prominence"[tiab] OR resonance*[tiab] OR dysphonia[tiab] OR laryngeal[tiab] OR larynx[tiab] OR laryn[tiab] OR communicat*[tiab] OR squeal*[tiab] OR babbl*[tiab]) | 00 |
| **#3: Keywords related to aging** |  |
| ("Aging"[Mesh] OR "Aging"[ tiab] OR "healthy aging"[tiab] OR "healthy ageing"[tiab] OR elderly[tiab] OR "older adults"[tiab] OR "older people"[tiab] OR geriatric*[tiab]) | 00 |
| **#4: Keywords related to diseases of old age with voice recognition capability** |  |
| **Neurodegenerative Disorders:** ("Alzheimer Disease"[ tiab] OR "Parkinson Disease"[ tiab] "cognitive impairment"[ tiab] OR "Dementia"[ tiab])  **Depression:** Depression [tiab] |  |
| #5: #1 AND #2 AND #3AND #4 AND | 00 |
