# Supplementary material for: Vocal biomarkers for geriatric health assessment: a scoping review protocol
Source: BMJ Open. 2026 Feb 23;16(2):e109480. doi: 10.1136/bmjopen-2025-109480 (PMC12931537; doi:10.1136/bmjopen-2025-109480)
Supplement: Supplementary file 3 [file bmjopen-2025-109480.reviewer_comments.pdf]

PEER REVIEW HISTORY

BMJ Open publishes all reviews undertaken for accepted manuscripts. Reviewers are asked to complete a checklist review form and are provided with free text boxes to elaborate on their assessment. These free text comments are reproduced below.

ARTICLE DETAILS

Title (Provisional)

Vocal Biomarkers for Geriatric Health Assessment: A Scoping Review Protocol

Authors

Amir-Behghadami, Mehrdad; gholipour, kamal; mohammadzadeh, Zaynab

---

VERSION 1 - REVIEW

**Reviewer**	**1**
**Name**	**Cury, Patricia**
**Affiliation**	**Universidade de São Paulo Faculdade de Medicina**
**Date**	**15-Oct-2025**
**COI**	**None**

This is a very interesting scope review . However, a few points should be improved:

* Abstract: Consider putting the sentences in the present, as the study was already done

* In pages 15 and 16, Ethical information is repeated. Only once would be enough.

* Discussion: A table with some of the results from the studies would be necessary to facilitate understanding.

**Reviewer**	**2**
**Name**	**Hudon, Alexandre**
**Affiliation**	**Université de Montréal**
**Date**	**09-Nov-2025**
**COI**	**None**

Brief summary

This manuscript, Vocal Biomarkers for Geriatric Health Assessment: A Scoping Review Protocol, presents a protocol for systematically mapping evidence on the use of artificial intelligence (AI) and machine learning (ML) in voice analysis for detecting and managing age-

related diseases. It follows PRISMA-ScR and Arksey & O'Malley frameworks, with a clear PCC structure and a comprehensive search strategy spanning 2012–2025. The topic is timely and relevant, addressing the intersection of aging, AI, and non-invasive diagnostics. Please see my comments below.

Introduction

• The introduction provides a comprehensive and well-referenced background linking global aging trends, limitations of traditional diagnostics, and the promise of AI-driven voice biomarkers.

• The justification for the review is strong, but the text could be streamlined to avoid redundancy (for example repeated Parkinson's disease examples).

• The rationale for including multiple disease domains is sound, though the clinical versus technical focus could be more clearly distinguished.

• Ethical considerations (particularly algorithmic bias, privacy, and inclusivity in geriatric populations) are appropriately raised but could be expanded in scope (as an example: governance frameworks).

• The section effectively sets the stage for a scoping review but would benefit from a clearer articulation of expected knowledge gaps and how findings will inform future research design or clinical adoption.

Methodology

• The use of PRISMA-ScR, Arksey & O'Malley, and JBI guidelines demonstrates high methodological rigor consistent with BMJ Open expectations for protocols.

• The PCC framework (Population, Concept, Context) is well structured; however, justification for the chosen age threshold (≥60 vs. ≥65 years) could be clarified to align with standard geriatric definitions.

• The inclusion/exclusion criteria are logically organized, but some boundaries (mixed-age studies, comorbid non-neurological voice disorders) could be operationalized more precisely to facilitate reproducibility.

• The extensive list of databases (including Persian-language sources) enhances coverage, though the feasibility of dual-language screening should be discussed.

• The data extraction plan is clear, but specifying how inter-rater reliability will be ensured (Cohen's κ, percent agreement) would improve transparency.

• The consultation stage is a valuable addition but should clarify the process for integrating stakeholder feedback (clinicians, AI engineers) into the final synthesis.

Discussion

• The discussion convincingly argues for the significance of synthesizing AI-driven voice biomarker evidence across geriatric conditions, moving beyond disease-specific reviews.

• The articulation of potential clinical and policy impacts is strong, but practical considerations (such as implementation in low-resource settings or the role of interdisciplinary training) could be elaborated.

• It would be useful to clarify how the authors plan to handle rapid technological evolution; inclusion of studies using generative or multimodal AI models after 2025.

• The limitations section correctly acknowledges the absence of quality assessment but could expand on potential publication bias and the evolving regulatory landscape for digital biomarkers.

VERSION 1 - AUTHOR RESPONSE

Reviewer: 1

Dr. Patricia Cury, Universidade de São Paulo Faculdade de Medicina, FACERES

Comments to the Author:

This is a very interesting scope review. However, a few points should be improved:

Authors response:

We also acknowledge your suggestion that certain points should be improved and are eager to receive your detailed feedback. Your insights will be invaluable in enhancing the rigor and quality of our work. We look forward to addressing your specific comments to strengthen the manuscript further.

* Abstract: Consider putting the sentences in the present, as the study was already done

Authors response:

Thank you very much for your insightful comment regarding the verb tense in the abstract. Since this manuscript is a protocol for an upcoming scoping review, we have used the future tense to clearly reflect that the study is planned and will be conducted later. This aligns with standard practice for protocol articles to convey the prospective nature of the work. We appreciate your suggestion and have carefully reviewed the abstract to ensure clarity and consistency while adhering to these conventions.
Thank you again for your valuable feedback.

* In pages 15 and 16, Ethical information is repeated. Only once would be enough.
Authors response:

Done.

* Discussion: A table with some of the results from the studies would be necessary to facilitate understanding.
Authors response:

Done.

Reviewer: 2

Dr. Alexandre Hudon, Université de Montréal

Comments to the Author:

Brief summary

This manuscript, Vocal Biomarkers for Geriatric Health Assessment: A Scoping Review Protocol, presents a protocol for systematically mapping evidence on the use of artificial intelligence (AI) and machine learning (ML) in voice analysis for detecting and managing age-related diseases. It follows PRISMA-ScR and Arksey & O'Malley frameworks, with a clear PCC structure and a comprehensive search strategy spanning 2012–2025. The topic is timely and relevant, addressing the intersection of aging, AI, and non-invasive diagnostics. Please see my comments below.

Authors response:

Thank you for reviewing our manuscript and for your positive and encouraging comments. We have addressed each of your comments point by point in detail. We hope that our comprehensive responses meet your expectations and contribute positively to improving our work.

Comments to the Author:

Introduction

• The introduction provides a comprehensive and well-referenced background linking global aging trends, limitations of traditional diagnostics, and the promise of AI-driven voice biomarkers.

• The justification for the review is strong, but the text could be streamlined to avoid redundancy (for example repeated Parkinson's disease examples).

• The rationale for including multiple disease domains is sound, though the clinical versus technical focus could be more clearly distinguished.

• Ethical considerations (particularly algorithmic bias, privacy, and inclusivity in geriatric populations) are appropriately raised but could be expanded in scope (as an example: governance frameworks).

• The section effectively sets the stage for a scoping review but would benefit from a clearer articulation of expected knowledge gaps and how findings will inform future research design or clinical adoption.

Authors response:

Thank you for your valuable feedback. In response to your requests, we have revised the introduction by streamlining the text to reduce redundancy, particularly by removing repeated examples of Parkinson's disease. We have clarified the distinction between the clinical focus and the technical focus of the study to enhance clarity. Furthermore, we have expanded the section on ethical considerations to include governance frameworks that ensure transparency, accountability, and inclusivity in the application of AI to eldercare. We believe these changes address your comments comprehensively and have improved the manuscript accordingly.

We appreciate your insightful suggestions which have strengthened our work.

Methodology

Comment to the Author:

The use of PRISMA-ScR, Arksey & O'Malley, and JBI guidelines demonstrates high methodological rigor consistent with BMJ Open expectations for protocols.

Authors response:

Thank you very much for your positive feedback. We appreciate your recognition of our adherence to PRISMA-ScR, Arksey & O'Malley, and JBI guidelines, which reflect our commitment to methodological rigor and align with BMJ Open expectations for systematic review protocols.

Comment to the Author:

The PCC framework (Population, Concept, Context) is well structured; however, justification for the chosen age threshold (≥60 vs. ≥65 years) could be clarified to align with standard geriatric definitions.

Authors response:

Thank you for your valuable comment. We have carefully reviewed the relevant literature and, as reflected in our references, the age threshold of 65 years and above is widely accepted as the standard definition for older adults in geriatric research. Accordingly, we have aligned our study criteria and abstract with this convention to ensure consistency and clarity.

Comment to the Author:

The inclusion/exclusion criteria are logically organized, but some boundaries (mixed-age studies, comorbid non-neurological voice disorders) could be operationalized more precisely to facilitate reproducibility.

Authors response:

Thank you for your valuable feedback. We acknowledge the importance of precise operationalization of inclusion and exclusion criteria to enhance reproducibility and clarity. To address this, we have refined the boundaries for mixed-age studies by specifying age ranges and have explicitly defined comorbid non-neurological voice disorders based on current clinical classifications distinguishing neurological versus non-neurological mechanisms (Toronto Speech Therapy, 2025). Specifically, we clarified that studies involving voice disorders primarily influenced by non-neurological conditions such as chronic obstructive pulmonary disease (COPD), laryngitis, gastroesophageal reflux disease (GERD), and acute infections like COVID-19-related voice changes are excluded to maintain focus on neurological etiologies. These refinements follow best practices for transparent systematic review criteria development to ensure reproducibility.

Comment to the Author:

The extensive list of databases (including Persian-language sources) enhances coverage, though the feasibility of dual-language screening should be discussed.

Authors response:

Thank you for your valuable feedback. We appreciate the importance of feasibility in conducting dual-language screening to ensure the rigor and reproducibility of our systematic review. We wish to clarify that all authors involved in this study are multilingual, proficient in Persian, Turkish, and English. This linguistic capability enables comprehensive screening across databases in these languages. Furthermore, the first author, who is fluent in all three languages, will conduct the screening process to maintain consistency and methodological rigor. These arrangements align with best practices for managing language diversity in systematic reviews, ensuring thorough coverage while maintaining high standards of accuracy and transparency (Wang et al., 2015; Cochrane Handbook, 2024). We will explicitly describe this process in the methods section for full transparency. The third step of selecting a study is explained on page 14.

Comment to the Author:

The data extraction plan is clear, but specifying how inter-rater reliability will be ensured (Cohen's κ, percent agreement) would improve transparency.

Authors response:

Thank you for your insightful comment regarding the specification of inter-rater reliability in our data extraction process. We agree that reporting measures such as Cohen's κ and percent agreement enhances transparency and methodological rigor. Accordingly, we have revised the manuscript to include that two researchers will independently review 20% of the studies and that inter-rater reliability will be quantitatively assessed using Cohen's κ and percent agreement before proceeding with single-researcher extraction. This approach ensures consistency and reliability in data extraction, as supported by best practices in systematic review methodology. The relevant text has been updated in the Data Charting section accordingly.

We appreciate your constructive feedback that has improved the clarity and robustness of our methods.

Comment to the Author:

The consultation stage is a valuable addition but should clarify the process for integrating stakeholder feedback (clinicians, AI engineers) into the final synthesis.

Authors response:

Thank you for your valuable comment. We have clarified that the consultation exercise in the final stage will initially be conducted internally and subsequently with a multidisciplinary expert panel including clinicians and AI engineers. Feedback collected will be systematically integrated into the synthesis to enhance the practical applicability of the clinical decision support system design. Please refer to pages 15-16, step six of the Arcey and O'Malley framework.

Comment to the Author:

Discussion

• The discussion convincingly argues for the significance of synthesizing AI-driven voice biomarker evidence across geriatric conditions, moving beyond disease-specific reviews.

• The articulation of potential clinical and policy impacts is strong, but practical considerations (such as implementation in low-resource settings or the role of interdisciplinary training) could be elaborated.

• It would be useful to clarify how the authors plan to handle rapid technological evolution; inclusion of studies using generative or multimodal AI models after 2025.

• The limitations section correctly acknowledges the absence of quality assessment but could expand on potential publication bias and the evolving regulatory landscape for digital biomarkers.The discussion has been expanded to address practical considerations in low-resource settings and interdisciplinary training. The protocol now clarifies plans for incorporating studies on generative and multimodal AI models beyond 2025. The limitations section includes potential publication bias and

acknowledges the evolving regulatory context for digital biomarkers. A new relevant reference has been added to support these points.

Authors response:

The discussion has been expanded to address practical considerations in low-resource settings and interdisciplinary training. The protocol now clarifies plans for incorporating studies on generative and multimodal AI models beyond 2025. The limitations section includes potential publication bias and acknowledges the evolving regulatory context for digital biomarkers. A new relevant reference has been added to support these points.

VERSION 2 - REVIEW

**Reviewer**	**2**
**Name**	**Hudon, Alexandre**
**Affiliation**	**Université de Montréal**
**Date**	**13-Dec-2025**
**COI**	

Brief summary

This manuscript presents a protocol for a scoping review aiming to map the use of AI driven vocal biomarkers in the detection and management of age related diseases among older adults. The topic is timely and relevant, and the protocol follows established scoping review frameworks. Please see my comments below.

Introduction: Potential improvements

• The introduction would benefit from a clearer and more concise articulation of the specific knowledge gap this scoping review seeks to address, beyond the general statement that the literature is fragmented.

• Several sections are descriptive and lengthy; tightening the narrative and reducing repetition would improve focus and readability.

• The justification for excluding non neurological conditions affecting voice could be clarified earlier to better align the scope with the stated aims.

• While ethical and implementation issues are mentioned, their relevance to the core review questions could be more explicitly linked.

Methods: Potential improvements

• The search strategy is extremely broad; further justification is needed to explain how feasibility and manageability of screening will be ensured.

• The rationale for including studies up to September 2025 should be clarified, particularly given the protocol publication timeline.

• The consultation exercise described in Stage 6 appears to extend beyond a traditional scoping review and would benefit from clearer boundaries and objectives.

• Data charting and synthesis plans are described in general terms; more specificity regarding how heterogeneity will be handled would strengthen methodological transparency.

Results: Potential improvements

• As this is a protocol, the manuscript would benefit from clearer guidance on the anticipated structure of the results to help readers understand how findings will be organized.

• The planned use of conceptual models and charts is noted, but examples or clearer descriptions of their purpose would improve clarity.

• It may be helpful to specify how overlapping disease domains or multimorbidity in older adults will be represented in the results.

• Consider clarifying how performance metrics across heterogeneous AI models will be summarized without implying comparative effectiveness.

Discussion: Potential improvements

• The discussion occasionally reads as if results are already known; this section should be more clearly framed as anticipated contributions rather than inferred findings.

• Claims regarding clinical and policy impact should be tempered to reflect the exploratory nature of a scoping review.

• Limitations specific to protocol design, such as language restrictions and exclusion of quality appraisal, could be more explicitly discussed.

• The final paragraphs could be streamlined to better distinguish between the purpose of the protocol and the expected implications of the completed review.

VERSION 2 - AUTHOR RESPONSE

Reviewer: 2

Dr. Alexandre Hudon, Université de Montréal

Comments to the Author:

Brief summary

This manuscript presents a protocol for a scoping review aiming to map the use of AI driven vocal biomarkers in the detection and management of age related diseases among

older adults. The topic is timely and relevant, and the protocol follows established scoping review frameworks. Please see my comments below. Authors response:

Comments to the Author:

Introduction: Potential improvements

• The introduction would benefit from a clearer and more concise articulation of the specific knowledge gap this scoping review seeks to address, beyond the general statement that the literature is fragmented.

• Several sections are descriptive and lengthy; tightening the narrative and reducing repetition would improve focus and readability.

• The justification for excluding non-neurological conditions affecting voice could be clarified earlier to better align the scope with the stated aims.

• While ethical and implementation issues are mentioned, their relevance to the core review questions could be more explicitly linked.

Authors response:

Thank you for your insightful comments on the introduction. We have carefully attended to your suggestions by articulating the specific knowledge gap more precisely, streamlining the narrative for conciseness, clarifying the exclusion of non-neurological conditions early to align with our aims, and explicitly linking ethical/implementation issues to the core review questions.

Comments to the Author:

<u>Methods: Potential improvements</u>

• The search strategy is extremely broad; further justification is needed to explain how feasibility and manageability of screening will be ensured..

Authors response:

Thank you for your valuable feedback on the search strategy. We have carefully considered your concerns regarding its breadth and have refined it accordingly to ensure feasibility and manageability in screening.

The finalized PubMed strategy now yields a reasonable number of records, maintaining consistency with the review's limited scope—focusing exclusively on neurological conditions (e.g., cognitive decline, Parkinson’s disease, Alzheimer’s, dementia, and depression) while excluding non-neurological voice-affecting conditions. Specifically, we have removed keyword groups for respiratory/cardiovascular issues (e.g., COPD, heart failure, stroke, aphasia), frailty/sarcopenia, and musculoskeletal disorders (e.g., osteoporosis, arthritis), aligning with the clarified aims in the revised introduction and avoiding dilution from irrelevant domains.

The finalized PubMed strategy now yields a reasonable number of records

("Artificial Intelligence"[Mesh] OR "artificial intelligence"[tiab] OR "machine learning"[tiab] OR "deep learning"[tiab] OR "computational intelligence"[tiab] OR "computer reasoning"[tiab] OR "computer vision system"[tiab] OR "computer vision systems"[tiab] OR "transfer learning"[tiab] OR "hierarchical learning"[tiab] OR "learning from labeled data"[tiab] OR "support vector network"[tiab] OR "support vector networks"[tiab] OR "support vector machine"[tiab] OR "support vector machines"[tiab] OR "ambient intelligence"[tiab] OR "automated reasoning"[tiab] OR "computer heuristics"[tiab] OR "cognitive technology"[tiab] OR "cognitive technologies"[tiab] OR "cognitive computing"[tiab] OR "cognitive robotics"[tiab] OR "optical character recognition"[tiab] OR "robotic process automation"[tiab] OR "machine intelligence"[tiab] OR "artificial superintelligence"[tiab] OR "artificial general intelligence"[tiab] OR "machine reasoning"[tiab] OR "automated inference"[tiab] OR "heuristic algorithm"[tiab] OR "heuristic algorithms"[tiab] OR metaheuristic*[tiab] OR meta-heuristic*[tiab] OR "data mining"[tiab] OR "neural network"[tiab] OR "neural networks"[tiab] OR "neural networking"[tiab] OR "feature learning"[tiab] OR "feature extraction"[tiab] OR "Bayesian learning"[tiab] OR "Bayesian inference"[tiab] OR "multicriteria decision analysis"[tiab] OR "unsupervised learning"[tiab] OR "semi-supervised learning"[tiab] OR "semi supervised learning"[tiab] OR "ANN analysis"[tiab] OR "ANN analyses"[tiab] OR "ANN method"[tiab] OR "ANN methods"[tiab] OR "ANN model"[tiab] OR "ANN models"[tiab] OR "ANN modeling"[tiab] OR "ANN methodology"[tiab] OR "ANN methodologies"[tiab] OR "artificial NN"[tiab] OR "ANN technique"[tiab] OR "ANN techniques"[tiab] OR "ANN output"[tiab] OR "ANN outputs"[tiab] OR "ANN approach"[tiab] OR "network learning"[tiab] OR "random forest"[tiab] OR "relevance vector machine"[tiab] OR "relevance vector machines"[tiab] OR "online analytical processing"[tiab] OR "sentiment analysis"[tiab] OR "sentiment analyses"[tiab] OR "opinion mining"[tiab] OR "sentiment classification"[tiab] OR "sentiment classifications"[tiab] OR "fuzzy logic"[tiab] OR "natural language processing"[tiab] OR "expert system"[tiab] OR "expert systems"[tiab] OR "biological ontology"[tiab] OR "biological ontologies"[tiab] OR "biomedical ontology"[tiab] OR "biomedical ontologies"[tiab] OR "computer simulation"[tiab] OR "computer simulations"[tiab] OR "Multidimensional Voice Program"[tiab] OR MDVP[tiab] OR "k-nearest neighbor"[tiab] OR "supervised learning algorithm"[tiab] OR "swarm intelligent"[tiab] OR "Swarm intelligence"[tiab] OR "firefly algorithm"[tiab] OR bootstrap*[tiab]) AND (voice[Mesh] OR voice*[tiab] OR speech*[tiab] OR acoustic*[tiab] OR phonat*[tiab] OR vox[tiab] OR language*[tiab] OR linguistic*[tiab] OR speak*[tiab] OR sing*[tiab] OR vocal*[tiab] OR respirat*[tiab] OR articulat*[tiab] OR prosody[tiab] OR pitch[tiab] OR "fundamental frequency*"[tiab] OR f0[tiab] OR "disturbance index"[tiab] OR jitter*[tiab] OR shimmer*[tiab] OR "vocal intensity"[tiab] OR "acoustic voice quality index"[tiab] OR AVQI[tiab] OR "speech to noise ratio*"[tiab] OR "sound pressure level*"[tiab] OR "cepstral peak prominence"[tiab] OR resonance*[tiab] OR dysphonia[tiab] OR laryngeal[tiab] OR larynx[tiab] OR laryn[tiab] OR communicat*[tiab] OR squeal*[tiab] OR babbl*[tiab]) AND ("Aging"[Mesh] OR "Aging"[tiab] OR "healthy aging"[tiab] OR "healthy ageing"[tiab] OR elderly[tiab] OR "older adults"[tiab] OR "older people"[tiab] OR geriatric*[tiab]) AND

(("Alzheimer Disease"[tiab] OR "Parkinson Disease"[tiab] "cognitive impairment"[tiab] OR "Dementia"[tiab]) OR Depression[tiab])

Comment to the Author:

<u>Methods: Potential improvements</u>

• The rationale for including studies up to September 2025 should be clarified, particularly given the protocol publication timeline.

Authors response:

Thank you for your insightful feedback on the search strategy timeline. We have carefully noted your concerns and revised the protocol accordingly.

The search will now be limited to studies up to the end of March 2026, aligning with the anticipated completion of the review process following protocol publication and ensuring capture of the most recent evidence without compromising feasibility.

Comment to the Author:

<u>Methods: Potential improvements</u>

• The consultation exercise described in Stage 6 appears to extend beyond a traditional scoping review and would benefit from clearer boundaries and objectives.

Authors response:

Thank you for your perceptive comments on the consultation exercise in Stage 6. We have taken note of your concerns regarding its scope and have made revisions to establish clearer boundaries and objectives, ensuring alignment with traditional scoping review methodology.

Comment to the Author:

<u>Methods: Potential improvements</u>

• The consultation exercise described in Stage 6 appears to extend beyond a traditional scoping review and would benefit from clearer boundaries and objectives.

Authors response:

Thank you for your astute observation on the data charting and synthesis plans. We have taken your comments into account and introduced greater specificity on handling heterogeneity, enhancing methodological transparency while preserving the scoping review's exploratory nature.

Comment to the Author:

<u>Results: Potential improvements</u>

Comment to the Author:

• As this is a protocol, the manuscript would benefit from clearer guidance on the anticipated structure of the results to help readers understand how findings will be organized.

• The planned use of conceptual models and charts is noted, but examples or clearer descriptions of their purpose would improve clarity.

• It may be helpful to specify how overlapping disease domains or multimorbidity in older adults will be represented in the results.

• Consider clarifying how performance metrics across heterogeneous AI models will be summarized without implying comparative effectiveness.

Authors response:

Thank you for your thoughtful suggestions on the results section. We have addressed each point systematically: clarifying the anticipated results structure by disease domain; specifying purposes and examples for conceptual models/charts; detailing representation of multimorbidity/overlaps; and emphasizing descriptive summary of heterogeneous performance metrics without comparative implications. **Please refer to the revised descriptions of Stages 4 and 5 of the Arksey and O'Malley framework (pages 13-14) for the specific changes implemented.**

Comment to the Author:

<u>Discussion: Potential improvements</u>

Comment to the Author:

• The discussion occasionally reads as if results are already known; this section should be more clearly framed as anticipated contributions rather than inferred findings.

• Claims regarding clinical and policy impact should be tempered to reflect the exploratory nature of a scoping review.

• Limitations specific to protocol design, such as language restrictions and exclusion of quality appraisal, could be more explicitly discussed.

• The final paragraphs could be streamlined to better distinguish between the purpose of the protocol and the expected implications of the completed review.

Authors response:

Thank you for your constructive feedback on the discussion section. We have addressed each point methodically: (1) reframing content to emphasize anticipated contributions rather than implying known results; (2) tempering clinical/policy impact claims to reflect the scoping review's exploratory scope; (3) explicitly discussing protocol-specific limitations like language restrictions and exclusion of quality appraisal; and (4) streamlining final paragraphs to distinguish protocol purpose from expected review implications.

VERSION 3 - REVIEW

**Reviewer**	**2**
**Name**	**Hudon, Alexandre**
**Affiliation**	**Université de Montréal**
**Date**	**18-Jan-2026**
**COI**	

The authors responded to all my previous comments. I consider this protocol to be scientifically valid and of quality.
